# No influence of the polymorphisms *CYP2C19 *and *CYP2D6 *on the efficacy of cyclophosphamide, thalidomide, and bortezomib in patients with Multiple Myeloma

**DOI:** 10.1186/1471-2407-10-404

**Published:** 2010-08-04

**Authors:** Annette J Vangsted, Karen Søeby, Tobias W Klausen, Niels Abildgaard, Niels F Andersen, Peter Gimsing, Henrik Gregersen, Ulla Vogel, Thomas Werge, Henrik B Rasmussen

**Affiliations:** 1 Dept. of Oncology and Haematology, Roskilde Hospital, Copenhagen University, DK-4000 Roskilde, Denmark; 2 Dept. of Clinical Biochemistry, Hvidovre University Hospital, 2650 Hvidovre, Denmark; 3 Dept. of Haematology, University Hospital of Copenhagen at Herlev, DK-2730 Herlev, Denmark; 4 Dept. of Haematology, Odense University Hospital, DK-5000 Odense, Denmark; 5 Dept. of Haematology, Aarhus University Hospital, DK-8000 Aarhus, Denmark; 6 Dept. of Haematology, University Hospital of Copenhagen at Rigshospitalet, DK-2100 Copenhagen, Denmark; 7 Dept. of Haematology, Aalborg University Hospital, DK-9100 Aalborg, Denmark; 8 National Food Institute, Technical University of Denmark, DK-2860 Copenhagen, Institute for Science, Systems and Models, Roskilde University, DK-4000 Roskilde, Denmark and National Research Centre for the Working Environment, DK-2100 Copenhagen, Denmark; 9 Research Institute of Biological Psychiatry, Mental Health Center Sct. Hans, University Hospital of Copenhagen, DK-4000 Roskilde, Denmark

## Abstract

**Background:**

The response to treatment varies among patients with multiple myeloma and markers for prediction of treatment outcome are highly needed. Bioactivation of cyclophosphamide and thalidomide, and biodegradation of bortezomib, is dependent on cytochrome P450 metabolism. We explored the potential influence of different polymorphisms in the CYP enzymes on the outcome of treatment.

**Methods:**

Data was analyzed from 348 patients undergoing high-dose treatment and stem cell support in Denmark in 1994 to 2004. Clinical information on relapse treatment in 243 individual patients was collected. The patients were genotyped for the non-functional alleles CYP2C19*2 and CYP2D6*3, *4, *5 (gene deletion), *6, and *CYP2D6 *gene duplication.

**Results:**

In patients who were treated with bortezomib and were carriers of one or two defective *CYP2D6 *alleles there was a trend towards a better time-to-next treatment. We found no association between the number of functional *CYP2C19 *and *CYP2D6 *alleles and outcome of treatment with cyclophosphamide or thalidomide. Neither was the number of functional *CYP2C19 *and *CYP2D6 *alleles associated with neurological adverse reactions to thalidomide and bortezomib.

**Conclusion:**

There was no association between functional *CYP2C19 *and *CYP2D6 *alleles and treatment outcome in multiple myeloma patients treated with cyclophosphamide, thalidomide or bortezomib. A larger number of patients treated with bortezomib are needed to determine the role of *CYP2D6 *alleles in treatment outcome.

## Background

The response to anticancer drugs varies considerably between patients, suggesting that individualized treatment might improve the response [1]. The individual differences in the efficacy of many drugs may reflect that genetic variations and genotype-based drug prescription represents a promising future means of individualizing drug treatment. Notably, genetic polymorphisms in enzymes implicated in drug metabolism represent a source of individual variation in drug responses. Among the drug metabolizing enzymes, the cytochrome P450 enzymes, such as CYP2C19 and CYP2D6, play a particularly important role [2].

Cyclophosphamide and thalidomide are used for the treatment of multiple myeloma and other cancers. The therapeutic effect is dependent on metabolic activation by CYP enzymes. Several CYP450 enzymes are implicated in the activation of cyclophosphamide including CYP2C19 [3]. This enzyme may play an even more significant role in the activation of thalidomide [4]. Bortezomib is a therapeutic proteasome inhibitor used for treatment of multiple myeloma (MM) and lymphoma. In contrast to cyclophosphamide and thalidomide, the activity of bortezomib is not dependent on biotransformation. The primary route of metabolism of bortezomib is oxidative deboronation by CYP2C19, CYP1A2, and CYP3A4 with contribution from CYP2D6 and several other CYP450 enzymes [5,6]. This abolishes the effect of bortezomib since the boron atom is necessary for the inhibition of the 26 S proteasome.

High-dose treatment with stem cell support (HDT) is standard treatment of younger patients with MM. Stem cells are harvested at regeneration following treatment with high-dose cyclophosphamide and granulocyte colony-stimulating factor. At recurrence of disease after HDT, new effective modalities such as thalidomide and bortezomib are now available and incorporation of these drugs has improved overall survival (OS) [7]. In Denmark, thalidomide was introduced as treatment at relapse of MM in 2000 and bortezomib was introduced for compassionate use in 2002.

Significant differences in the outcome of treatment of MM are observed. This variation can be explained by biological aspects of the tumour, tumour burden at diagnosis, immune response and by differences in response to therapy caused by genetic variations in the drug metabolizing enzymes or genes involved in DNA repair or inflammation [8-10].

In this study, we analyzed the influence of commonly occurring functional polymorphisms in *CYP2C19 *and *CYP2D6 *on treatment outcome in relation to high-dose cyclophophamide and treatment with thalidomide or bortezomib at recurrence of disease.

## Methods

Subjects, clinical data, response criteria, and eligibility criteria have previously been described in detail [9-11]. Briefly, patients diagnosed with MM, and treated with high-dose melphalan and stem cell support from August 1994 to August 2004, were recruited from four participating centres in Denmark. Peripheral blood stem cells were harvested at regeneration after cyclophosphamide priming and G-CFS, and the patients hereafter underwent high-dose chemotherapy with melphalan (200 mg/m^2^) followed by stem cell support. Information on treatment at relapse was obtained from the medical reports from February to May 2008.

Staging was according to Durie and Salmon and the International Staging System (ISS). Time-to-treatment failure (TTF) and OS were calculated from date of stem cell infusion to date of progression or death, respectively. TTF was used as follow-up after HDT. Time-to-next treatment (TNT) was used as follow-up after relapse treatment and was defined as the period from start of relapse treatment to start of new relapse treatment, progression without new treatment or death. Duration of treatment was from initiation of relapse treatment to cessation of treatment.

The retrospective collection of data allowed analyzing response to treatment as 1) partial response or more and 2) no change or progressive disease. Partial response was defined by a reduction of at least 50% in the initial serum M-protein concentration and a reduction of light chain proteinuria to less than 0.2 g/24 h. No change was defined as not meeting the criteria of partial response or progression. Progression was defined by a more than 25% increase in serum M-protein or 25% increase in immunoglobulin levels above upper normal levels, confirmed by 2 separate measurements at least one month apart. Increase in bone marrow infiltration of plasma cells by 25%, increase of Bence-Jones proteinuria to more than 1.0 g/24 hr or other signs of progression such as hypercalcemia, progressive skeletal disease or soft-tissue plasmacytoma were also considered as progression. The occurrence of other malignancies and death without progression was regarded as events not related to progression. These patients were included in the analysis of OS.

Adverse reactions were noted as peripheral neuropathy caused by treatment with thalidomide or bortezomib. The degree of neurological adverse reactions were summarized as follows: level 1; no dose reduction at treatment, level 2; dose reduction due to neurological adverse reactions and level 3; cessation of treatment due to neurological adverse reactions.

The present study was approved by the Danish Ethical Committee (01-158/03).

### Statistical methods

SPSS statistical software was used for all calculations (SPSS for Windows, Rel. 14.0.0. 2005, Chicago: SPSS Inc.) and R statistical software, version 2.9.2 (R Foundation for Statistical Computing, 2009, Vienna, Austria). All tests were two-sided and p-values < 0.05 were regarded as statistically significant. Fisher's exact test was used for comparing categorical variables and Mann-Whitney test was used to detect differences in the distribution of continuous variables. The Kaplan-Meier method and the log rank test were used to compare TTF and OS between groups. For OS and TTF, Cox proportional hazard model was used for adjustment for prognostic factors. For treatment duration, linear regression analysis was used for adjustment for prognostic factors. For treatment response, logistic regression analysis was used for adjustment for prognostic factors. Statistical corrections for multiple testing were not carried out since none of the p values in our study reached significance at the 5% level. In power calculation the sample size and hazard ration (HR) were calculated with a power of 80% and a two-sided level of significance at 0.05. Power calculations were based on the proportion of events found in this study.

### DNA purification

DNA for analysis was purified from peripheral blood mononuclear cells by the salting out method [12] or from paraffin embedded tissue by phenol extraction as described elsewhere [13].

### Genotyping of single nucleotide polymorphisms

Determination of *CYP2D6 *gene deletion and duplication was based upon long distance PCR determination of *CYP2D*6 gene deletion [14] and duplication [15]. Typing of the remaining polymorphisms was carried out using commercially available 5'-exonuclease dependent assays with proprietary amplification primers and allele-specific fluorescent labeled probes. (Applied Biosystems, Foster City, CA, USA).

Patients were categorized according to their number of functional alleles; *CYP 2C19*: absence of functional alleles, poor metabolizers (PM); one functional allele, intermediate metabolizers (IM) and two normal alleles, extensive metabolizers with normal capacity of the enzyme (EM). A similar approach was used for classification of *CYP2D6 *genotypes with the supplementation of an additional class consisting of genotypes with more than two functional *CYP2D6 *genes reflecting duplication, ultra rapid metabolizers (UM).

## Results

Three-hundred and forty-eight patients were treated with HDT and stem cell support. The median follow-up of all patients still alive was 93.4 months (54.6-174.2 months). The median OS was 69.8 months (60.5-81.6 months). The median TTF was 27.7 months (23.4 to 30.8 months). Two-hundred and forty-three patients suffered from relapse which required treatment. The median follow-up time of patients with relapse was 91.4 months (60-158.8 months) and the median OS after HDT was 56.3 months. The genotype distributions of *CYP2C19 *and *CYP2D6 *in relation to prognostic markers are presented in table 1. In *CYP2C19 *a difference in creatinine levels (p = 0.03) and ISS stage (p = 0.04), was found between EM and IM + PM. In *CYP2D6 *a difference was found in sex in relation to UM + EM and IM + PM (p = 0.04).

**Table 1:** Characteristics of the patient population subdivided by the polymorphisms in *CYP2C19 *and *CYP2D6*.

	*CYP2C19* EM	*CYP2C19* IM + PM	P value	*CYP2D6* UM + EM	*CYP2D6* IM + PM	P value
Age	55 (28-69)	57 (31-66)	0.80	56 (28-69)	56 (35-68)	0.5

β2-micro-globulin	3.7 (1.2-55.6)	4.9 (1.5-22.0)	0.09	4.1 (1.3-36.0)	3.7 (1.2-56.6)	0.9

Creatinine	1.1 (0.6-9.4)	1.3 (0.5-8.4)	0.03	1.1 (0.5-9.4)	1.1 (0.7-6.9)	0.8

Albumin	3.5 (0.3-5.3)	3.3 (1.6-5.3)	0.07	3.5 (0.3-5.2)	3.5 (0.3-5.0)	0.5

Durie-Salmon Stage						
I	29 (11%)	5 (7%)	0.4	10 (8%)	11 (11%)	0.5
II	62 (24%)	14 (19%)		25 (20%)	23 (23%)	
III	167 (65%)	54 (74%)		89 (72%)	64 (65%)	

ISS						
I	46 (25%)	8 (16%)	0.04	18 (22%)	21 (28%)	0.5
II	78 (42%)	15 (31%)		33 (41%)	24 (32%)	
III	60 (33%)	26 (53%)		30 (37%)	29 (39%)	

Sex						
male	149 (56%)	48 (66%)	0.2	68 (54%)	68 (67%)	0.04
female	116 (44%)	25 (34%)		59 (46%)	33 (33%)	

**Figure 1:**
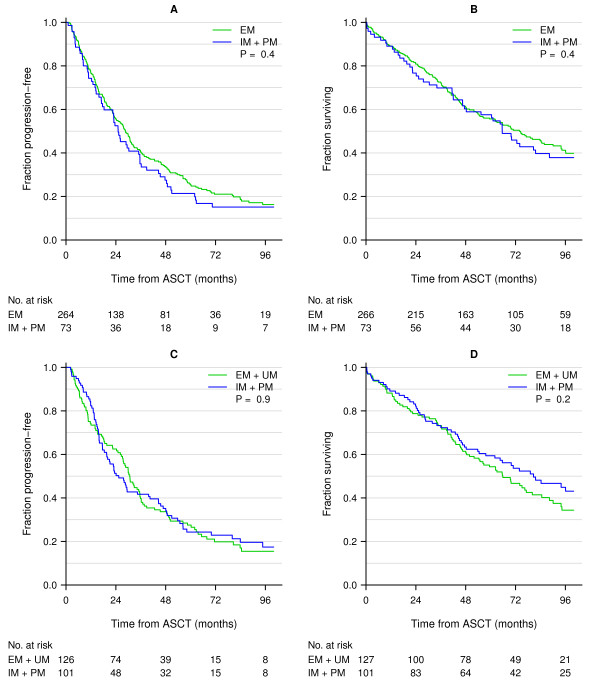
**Kaplan-Meier plots of TTF and OS in all patients treated with high-dose treatment in relation to the *CYP2C19 *(A) and *CYP2D6 *(B) polymorphisms**. The numbers at risk at 0, 24, 48, 72 and 96 months are presented below the figure. A: The green line represents extensive metabolizers (EM) and the blue line represents intermediate metabolizers (IM) and poor metabolizers (PM). B: The green line represents EM and ultra rapid metabolizers (UM) and the blue line represents patients with IM and PM status.

**Figure 2:**
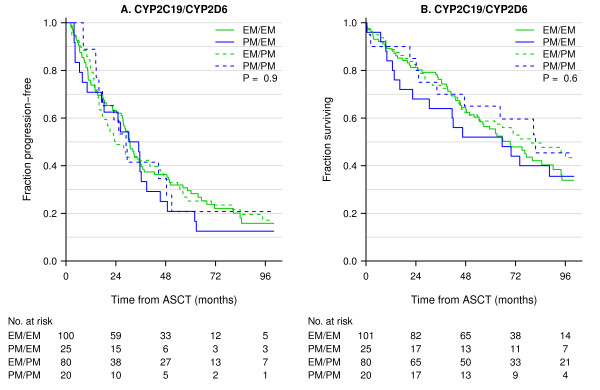
**Kaplan-Meier plots of TTF and OS in all patients treated with high-dose treatment using combinations of genotypes *CYP2C19 *and *CYP2D6***. The numbers at risk at 0, 24, 48, 72 and 96 months are presented below the figure. 1) EM for *CYP2C19 *and EM for *CYP2D6 *(solid green line); 2) PM for *CYP2C19 *and EM for *CYP2D6 *(solid blue line); 3) EM for *CYP2C19 *and PM for *CYP2D6 *(dashed green line); 4) PM for *CYP2C19 *and PM for *CYP2D6 *(dashed blue line).

The study included 348 myeloma patients treated with high-dose treatment and stem cell support. The *CYP 2C19 *genotypes were determined in 339 patients and *CYP2D6 *in 228 patients. The allele frequency of CYP2C19*2 was 0.109. The allele frequencies of *CYP2D6 *alleles *3, *4, *5, *6 and *CYP2D6 *gene duplication were 0.013, 0.219, 0.038, 0.006 and 0.015, respectively. These frequencies are in accordance with data from other Northern and Western European populations [16]. Successful analysis and data of response after high-dose cyclophosphamide was available for 247 patients. No differences in response, TTF after HDT and OS was found (Table 2, Table 3 and Figure 1). Adjustment for prognostic markers such as age, sex, β2-microglobuloin, creatinine and Durie-Salmon stage did not influence response to cyclophosphamide treatment. In *CYP2C19*, 266 patients were EM and 73 patients were IM or PM. With this distribution, power calculations revealed that we had 80% chance of detecting an HR of 1.5 and 1.6 for TTF and OS, respectively. For *CYP2D6 *127 patients were UM or EM and 101 patients were IM or PM. With this distribution, power calculations revealed that we had 80% chance of detecting an HR of 1.5 and 1.6 for TTF and OS, respectively. Combinations of metabolizer status for in *CYP2C19 *and *CYP2D6 *were analysed for the effect of high-dose treatment with cyclophosphamide: 1) EM for *CYP2C19 *and EM for *CYP2D6*, 2) PM for *CYP2C19 *and EM for *CYP2D6*, 3) EM for *CYP2C19 *and PM for *CYP2D6*, 4) PM for *CYP2C19 *and PM for *CYP2D6*. There was no difference in outcome for patients carrying the various combinations of *CYP2C19 *and *CYP2D6 *(Figure 2).

**Table 2:** Analysis of the effects of phenotypes on TTF and OS.

Genotype	N	%	Median TTF (months)	p value	Median OS (months)	p value
***CYP2C19***						

EM	266	78	28.7	0.4 *(0.4)*	73.9	0.4 *(0.5)*

IM + PM	73	22	25.1		65.6	

**CYP2D6**						

UM + EM	127	56	30.7	0.9 *(0.8)*	65.9	0.2 *(0.4)*

IM + PM	101	44	25.4		80.7	

Values in italics are adjusted for prognostic-related factors (TTF: β2-microglobulin.

OS: β2-microglobulin, creatinine and Durie-Salmon stage) and are shown in parentheses.

**Table 3:** Phenotypes in *CYP2C19 *and *CYP2D6 *and outcome of treatment with cyclophosphamide, thalidomide and bortezomib.

Treatment	Genotypes classes	CR + PR (%)	NC + PD (%)	P value	Duration **of treatment**, months	P value	TTF^1^/TNT^2^ months	P value	OS months	P value
**Cyclophosphamide**	***CYP2C19***									
	EM vs.	164 (78)	46 (22)	0.7	NR	NR	28.7	0.4	73.9	0.4
	IM + PM	23 (75)	14 (24)				25.1	*(0.4)*	65.6	*(0.5)*

**Thalidomide**	***CYP2C19***									
	EM vs.	78 (62)	48 (38)	0.5	7.4	0.9	10.5	0.6	62.4	0.8
	IM + PM	22 (53)	18 (45)		7.0		7.4		65.6	

**Bortezomib**	***CYP2C19***									
	EM	36 (73)	13 (27)	0.5	3.8	0.6	8.8	0.9	83.4	0.9
	IM + PM	13 (27)	3 (13)	*(0.3)*		*(0.6)*	7.8	*(0.7)*	74.0	*(0.9)*

	***CYP2D6***									
	UM + EM	21 (81)	5 (19)	1	4.2	0.8	7.8	0.07	69.7	0.12
	IM + PM	16 (84)	3 (16)	*(0.9)*		*(0.9)*	11.3	*(0.2)*	96.1	*(0.2)*

NR, not relevant; EM, extensive metabolizer; IM, intermediate metabolizer; PM, poor metabolizer; UM, ultra rapid metabolizer;

CR, complete response; PR, partial response; NC, no change; PD, progressive disease. TTF, time-to-treatment failure;

TNT, time-to-next treatment

1) TTF is used for evaluation of efficacy of treatment with cyclophosphamide

2) TNT is used for evaluation of efficacy of treatment with thalidomide and bortezomib.

For treatment with cyclophosphamide values in italics are adjusted for prognostic markers: TTF: β2-microglobulin; OS: β2-microglobulin, creatinine and Durie-Salmon stage. At relapse age was a prognostic marker. No influence of age was found for patients treated with thalidomide. For treatment with bortezomib values in italics are adjusted for age at start of relapse treatment and are shown in parentheses.

At relapse 177 patients were treated with thalidomide and 74 patients were treated with bortezomib. Some patients treated with thalidomide were later treated with bortezomib. Clinical data and successful analysis were available for 166 patients treated with thalidomide and 65 patients treated with bortezomib (*CYP2C19 *(65 patients) and *CYP2D6 *(45 patients)). In this setting, age was a prognostic factor at relapse. In patients treated with thalidomide, age did not influence treatment outcome. However, in patients treated with bortezomib older age was associated with poor outcome (p = 0.04). Adjustment for age in patients treated with bortezomib did not change the results. For patients treated with thalidomide, we found no difference in response to treatment or TNT in carriers of defective *CYP 2C19 *and *CYP2D6 *alleles (Table 3). For *CYP2C19*, 78 patients were EM and 22 patients were IM or PM. With this distribution, power calculations revealed that we had 80% chance of detecting an HR of 1.7 and 1.8 for TTF and OS, respectively. Very few data were available for patients treated with bortezomib. A trend towards a better TNT was found for patients treated with bortezomib who were carriers of one or two defective *CYP2D6 *alleles, i.e. patients classified as IM and PM (p = 0.07). For patients treated with bortezomib the genotype distribution of *CYP2C19 *revealed that 36 patients were EM and 13 patients were IM or PM. With this phenotype distribution, power calculations showed that we had 80% chance of detecting a HR of 2.5 and 2.7 for TTF and OS, receptively. For *CYP2D6 *21 patients were UM and EM and 16 patients were IM or PM. With this phenotype distribution, we had 80% chance of detection a HR of 2.6 and 2.7 for TTF and OS, respectively. Finally, no association was found between CYP phenotypes and treatment with either thalidomide or bortezomib in relation to neurological adverse reactions (Table 4). Using the genotype distribution of *CYP2C19 *found in this study, power calculations showed that 283 events were required for significant findings for a HR of 1.5. With the proportion of events in our study, sample size should be 473 for OS and 359 for TTF. With a larger HR of 2.0, 97 events were required, leading to a sample size of 162 and 123 for TTF and OS, respectively. Similar calculations, using the distribution of *CYP2D6*, showed that a sample size of 194 events were required for a HR of 1.5 and a sample size of 324 for OS, and a sample size of 246 for TTF. With a HR of 2.0, 66 events were required which implies a sample size of 111 for OS and 85 for TTF.

**Table 4:** Neurologic adverse reactions of thalidomide and bortezomib in relation to phenotype in *CYP2C19 C *and *CYP2D6*

Parameter	*CYP2C19* EM (%)	*CYP2C19* IM + PM (%)	P value	*CYP2D6* UM + EM (%)	*CYP2D6* IM + PM (%)	p value
Thalidomide						
Grade 0 + 1	64 (51)	21 (52)	0.6	NR	NR	0.7
Grade 2	36 (29)	5 (13)				
Grade 3	26 (21)	14 (35)				

Bortezomib						
Grade 0 + 1	22 (46)	8 (50)	0.7	14 (54)	8 (42)	0.5
Grade 2	11 (23)	4 (25)		7 (27)	6 (32)	
Grade 3	15 (31)	4 (25)		5 (19)	5 (26)	

NR: not relevant

## Discussion

We examined the influence of genetic variation in genes encoding CYP2D6 and CYP2C19 on the outcome of treatment of MM patients with high-dose cyclophosphamide, thalidomide and bortezomib. A trend towards better outcome was observed for the *CYP2D6 *IM and PM phenotype status in response to treatment with bortezomib, perhaps reflecting a slower inactivation of this drug in patients with defective *CYP2D6 *alleles. *CYP2C19 *genotype status was neither associated with treatment outcome after high-dose cyclophosphamide nor with outcome of thalidomide treatment. Moreover, neurological adverse reactions upon treatment with thalidomide or bortezomib did not appear to be dependent upon *CYP2C19 *and *CYP2D6 *genotypes.

In contrast to our findings, a small Chinese study found that myeloma patients with *CYP 2C19 *PM genotype responded less well to treatment with thalidomide [4]. In our study, only one patient with two defective *CYP2C19 *alleles was found. Interestingly, this patient did not respond to treatment with thalidomide suggesting that patients lacking CYP2C19 activity may respond less well to treatment with thalidomide.

Other CYP enzymes than CYP2C19 and CYP2D6 are also of relevance in relation to outcome of treatment with cyclophosphamide, thalidomide and bortezomib. A recent study failed to detect associations between polymorphisms in *CYP3A4/CYP3A5 *and the outcome HDT in MM patients with HDT [17]. This may reflect that the selected polymorphisms do not change enzyme activity to any significant extent and that they are not in linkage disequilibrium with any functional *CYP3A4 *or *CYP3A5 *polymorphism.

There are limitations to the present study. Notably, a larger patient sample would have been desirable and permitted us more reliably to assess the strength of an association between *CYP2D6 *genotype status and efficacy of bortezomib treatment. With a larger patient sample it would probably also have been possible to validate the association between treatment with thalidomide and *CYP2C19 *genotype status detected among Chinese patients [4]. Several CYP enzymes are involved in metabolism of cyclophosphamide, thalidomide and bortezomib. It is therefore possible that design of individualized treatments require determination of additional genotypes [18].

## Conclusions

In conclusion, we found no association between *CYP2C19 *and *CYP2D6 *polymorphisms on treatment effect by cyclophosphamide, thalidomide and bortezomib in patients with multiple myeloma after HDT with stem cell support. However, a larger number of patients treated with bortezomib are needed to exclude the effect of *CYP2D6 *polymorphisms on treatment outcome.

## List of abbreviations

MM: multiple myeloma; OS: overall survival; TTF: time-to-treatment failure; TNT: time-to-next treatment; HDT: high-dose treatment with stem cell support; CYP: cytochrome P450; UM: ultra rapid metabolizers; EM: extensive metabolizers; IM: intermediate metabolizers; PM: poor metabolizers.

## Competing interests

The authors declare that they have no competing interests.

## Authors' contributions

Conception and design, analysis and interpretation of data, drafting the article, approval of the version to be published (AJV, HBR).

Conception and design, analysis and interpretation of data, approval of the version to be published (KS, UBV and TW).

Analysis and interpretation of data, revising the manuscript critically, and approval of the version to be published (TWK, NA, PG, and HG)

## Pre-publication history

The pre-publication history for this paper can be accessed here:

http://www.biomedcentral.com/1471-2407/10/404/prepub
